# What effect do point of care fees have on childhood consultations in general practice?

**DOI:** 10.1186/s12913-018-3800-8

**Published:** 2018-12-18

**Authors:** Andrew O’Regan, Walter Cullen, Clodagh O’Gorman, Louise Hickey, Eimear O’Neill, Jane O’Doherty, Ailish Hannigan

**Affiliations:** 1 Graduate Entry Medical School, University of Limerick, Castletroy, Limerick, Ireland; 2 University College Dublin, School of Medicine, Health Sciences Centre, Belfield, Dublin 4, Ireland

**Keywords:** Health planning, General practice, Healthcare systems, Primary healthcare, Paediatrics

## Abstract

**Background:**

General practice (GP) has historically been central to the prevention and treatment of childhood illnesses. In Ireland, this role has recently expanded with the introduction of free GP care for children aged under six years in 2015. The Republic of Ireland has the only health system in the European Union which does not offer universal coverage for primary care. This study aims to analyse general practice records to investigate the effect of point of care consultation fees on childhood attendances.

**Methods:**

GPs affiliated to the medical school (*n* = 72) were invited to participate. 100 children aged 1 to 14 years were randomly sampled from each. Data was collected on service utilisation in the previous 12 months, specifically: age, gender, eligibility for free care and whether they had consulted their GP in the 12 month period.

**Results:**

Sixty-four practices participated, producing data on 6007 eligible children. The median age of children was seven years; 3688(62%) were ‘fee-paying’. GMS patients aged under six years had a median of three consultations/year, with a quarter attending six times a year or more, while fee paying patients had a median of two consultations/year with a quarter attending four times a year or more.

**Conclusions:**

Children eligible for free care attend more often with a subgroup attending very frequently. This study provides important information on the possible impact of fees on healthcare utilisation for countries considering co-payment.

## Background

The majority of childhood illness in the western world is managed by general practitioners (GPs) [1], with most children in the UK attending their GP three to six times per year [2]. Acute cough alone in the 0–4 age group is estimated to cost the National Health Service in the UK £31.5 million per year, mainly through consultations in general practice [3]. The ratio of GPs to population in Ireland is 0.63 per 1000 [4], compared to 0.7 in the UK [5] and the European average of 0.8 per 1000 [6].With a rise in the number of GP attendances expected with demographic changes [2], it is recognised that there is a mismatch between demand for and supply of primary care services with ever increasing workloads, stress and burnout for GPs [6]. In response, some commentators, including the Australian Centre for Health Research, have proposed an ‘upfront fee’ for GP visits to reduce service utilisation and cut health spending [7]. The way in which payment is organised can influence health behaviour, increasing consultations with GPs and consequently clinical workload [8].

A recent paper by the British Medical Association describes several models of general practice payment worldwide [9]. Different payment systems exist and, in most European countries, a mixture of systems is in place. Fee-for-service predominate in Belgium, Denmark and France and involves payment calculated on work performed. Performance related payment is calculated based on GPs reaching set clinical targets. Capitation systems are commonplace involve a sum paid to the GP per patient for a period of time and are in place in the UK, Italy and Netherlands. These countries have universal health care systems where general practice visits are fully paid for by the state. On the other hand, some European countries such as Sweden use co-payment where fees are charged to supplement state payments but there is an annual ceiling on out of pocket expenses. Most systems are nuanced, such as Malta, where most of the population have access to state funded GP care but, compared to private general practice, it is limited in terms of the services and continuity of care that it provides [10]. There is considerable overlap between public and private general practice, whereby many people from all socio-economic groups pay to attend private GPs for a variety of cultural and social reasons [11].

In the Republic of Ireland, GPs are remunerated through a mixed public-private system. Private patients pay at the point of contact, usually at a cost of 50 euro for adults and 30 euro for children. For public patients, the state covers the costs of general practice care. The latter category comprises 43% of the population [12]. Qualification for publicly funded care is based on a means testing system, whereby patients from families that have a net income below a cut-off point are issued a GMS or doctor visit card and GPs are paid a per capita fee.

Major restructuring of the health service delivery has taken place since 2015, providing for free GP care to all children under 6 years in Ireland [13], with further expansion to under 12 year olds planned. Part of the strategy includes providing payment for GPs to conduct periodic wellness checks on children in order to “reorient the focus of primary care towards active health promotion” [14]. However, the restructuring has met much resistance from GPs because of the potential impact of free medical care for children on consultation rates and subsequent workload costs to general practice in Ireland [15].

In the context of current debate on co-payment [16], there is a unique opportunity to investigate the effect of fee-paying status on the health care utilisation of children aged 1–14 years. This study, which was conducted during the 12-months before restructuring, on a nationally representative sample aimed to compare consultation rates, reasons for presentation and actions documented by the GP during consultations.

## Methods

### Setting

A letter of invitation describing the study was sent to each practice that had a senior medical student on clinical placement in 2014/15 (*n* = 72 practices). The University of Limerick Graduate Entry Medical School is unique in the Irish medical education context in that it has a longitudinal integrated clerkship in general practice whereby students spend 18-weeks on clinical placement and take on roles and responsibilities within the practice setting. Ireland has four healthcare regions and the medical school has a network of practices that extends to three of the healthcare regions. Based on previous research, the practices involved with the medical school are generally comparable to the national profile in terms of practice characteristics and patient demographics [17].

### Participants

Under the supervision of their GP tutors, senior medical students on placement used the electronic practice management systems to generate a list of all patients aged 1 (to facilitate a review of one year of healthcare utilisation) to 14 years (the upper age for new referrals to paediatrics in many clinics). Using Microsoft Excel, the students were able to randomly select a sample of 100 children. Children who had not been registered with the practice for 12 months were excluded.

Data was collected on consultations that took place over a 12 month period between 1/09/13 to 31/08/14. This timeframe was before the legislation for free health care to children under-6 years was implemented.

### Measures

The clinical records for the sample of children over the 12 month period were reviewed by the senior medical student on placement and their supervising GP. All children had information collected on their age, gender, eligibility for free consultations and whether they had consulted their GP in the 12 month period. A consultation was defined as any visit to the practice or telephone conversation that resulted in an entry to the child’s records excluding attendances solely for the purpose of immunisations (these are funded separately by the State) and out of hours attendances which are resourced separately by out of hours GP co-operatives.

For those who had attended in the 12 month time period, information on the number of consultations in general practice in that time period, presenting symptoms at the most recent attendance (up to three per consultation), and actions documented by the GP (prescribe, reassure, refer, further investigations e.g. blood tests, X-Rays, preventative and health promotion advice and other) were recorded. Presenting symptoms were coded post hoc using the ICPC-2 coding system [18]. Anonymised datasets from all practices were merged into a database with practice characteristics (urban or rural, number of patients, number of staff). It was not possible to record socio-economic characteristics as they were not recorded on patient files.

### Statistical analysis

Numeric variables were tested for normality and summarised using mean (standard deviation) for normally distributed variables and median (first quartile, third quartile) for skewed distributions. Counts and percentages are presented for categorical data. Pearson’s chi-square test was used to test for significant associations between categorical variables. A 5% level of significance was used for all tests. Cramer’s V was used as a measure of the strength of the association, with 0.1 consider small, 0.3 medium and 0.5 large. A binary logistic regression analysis was carried out to predict visit in the last year (yes, no) using gender, age and eligibility for fee exemption medical care (not eligible, eligible) as predictor variables. All analysis was carried out using IBM SPSS Version 21.

Ethical approval for the study was granted by the Irish College of General Practitioners Research Ethics Committee (ICGP). The students involved in data collection were trained in the process by faculty and all data were de-identified at source and stored appropriately. Consent for use of clinical records was not required which was in line with the ICGP research guidelines at the time of the study.

## Results

Of the 72 practices affiliated with the medical school, 64 (89%) participated in the study. Practice size ranged from under 2000 to over 10,000 registered patients (registered number of children aged under fifteen years ranged from 200 to over 1000). Twenty eight (44%) practices indicated they were ‘urban’, eighteen (28%) indicated they were ‘rural’ and eighteen (28%) indicated they were ‘mixed’ practices. Children who had not been registered with the practice for 12 months were excluded from the sample with analysis based on data from 6007 patients aged 1 to 14 years from the 64 participating practices. The median age of the children was 7 years (first quartile = 4, third quartile = 11); 3083 (51%) were male and 3688 (62%) were fee-paying patients.

**Fig. 1:**
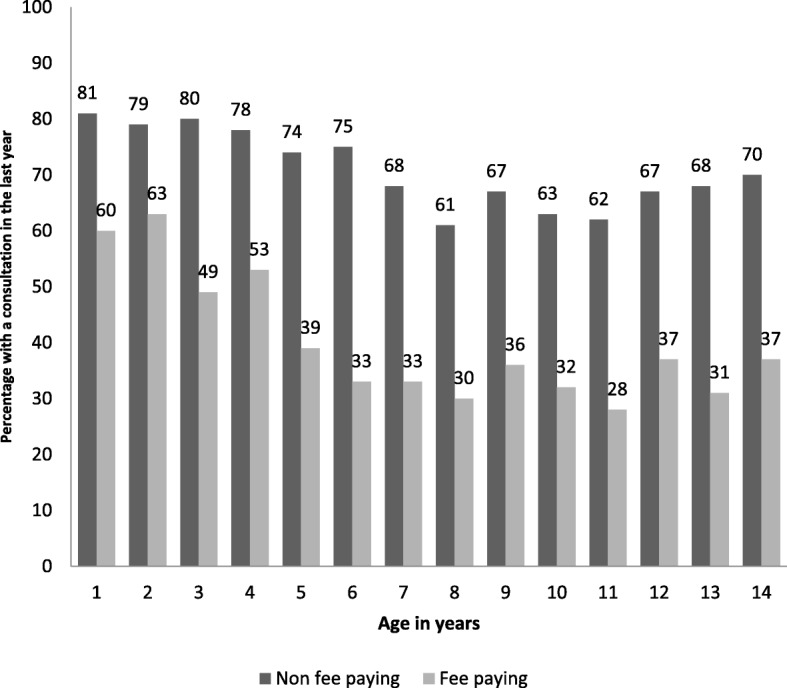
Percentage with a consultation in the last year by age in years and fee paying status

3105 (52%) of children in the sample had a consultation with their general practitioner within the last year (71% of those eligible for fee exemption consultations vs. 40% of fee-paying, *p* < 0.001, Cramer’s V = 0.30). Figure 1 summarises the percentage of children within each one year of age band with a visit in the last year by eligibility for fee-paying consultations. Those eligible for fee exemption were almost four times as likely to have a visit in the last year compared to fee-paying children (adjusted OR = 3.86, 95% confidence interval: 3.44 to 4.32).

There were 9583 consultations documented in total with the majority of these (62%) in children eligible for fee exemption consultations. Of the children in the sample with at least one visit to their GP in a 12-month period, the median number of consultations for those eligible for fee exemption consultations was three per year compared to two for fee-paying children (Table 1).

**Table 1:** Median number of GP consultations (first quartile, third quartile) by age group and fee paying status

Fee paying status	All children *n* = 6007	Children with at least one visit in past year *n* = 3105	All children under 6 *n* = 2191	Children under 6 with at least one visit in past year *n* = 1351
Fee paying	0 (0, 1)	2 (1, 3)	1 (0, 2)	2 (1, 4)
Eligible for fee exemption	2 (0, 4)	3 (1, 5)	2 (1, 5)	3 (2, 6)
All patients	1 (0, 2)	2 (1, 4)	1 (0, 3)	3 (1, 5)

Active patients eligible for fee exemption consultations and aged under six years had a median of three consultations within the 12-month time period with a quarter attending six times a year or more compared to a median of two for fee-paying patients with a quarter attending four times a year or more.

For children who had attended in the past year, the presenting symptoms documented at their most recent consultation are summarised in Table 2. The most common presenting symptom was respiratory (39%) in both fee paying and fee exemption children. There were no significant differences between the presenting complaints by fee paying status apart from those related to the ear with fee-paying children slightly more likely to present with symptoms in this category (Table 2).

**Table 2:** Presenting symptoms documented by the GP at the most recent consultation in the past year by fee paying status

Presenting Symptom (ICPC-2 category)	Fee paying (*n* = 1447)	Eligible for fee exemption (*n* = 1601)	*P*-value (Cramer’s V)
Respiratory	565 (39%)	616 (39%)	0.75 (0.006)
Skin	233 (16%)	291 (18%)	0.13 (0.03)
Digestive	143 (10%)	170 (11%)	0.50 (0.01)
General and unspecified	136 (9%)	141 (9%)	0.57 (0.01)
Ear	97 (7%)	79 (5%)	0.04 (0.04)
Musculoskeletal	80 (6%)	104 (7%)	0.26 (0.02)
Other	193 (13%)	200 (12%)	0.49 (0.01)

Table 3 summarises the actions documented by the GP at the child’s most recent attendance by fee-paying status. The most common actions documented by the GP were prescribing and reassurance. Fee-paying children were more likely to be prescribed medication than fee exemption children (62% vs 58%, *p* = 0.007, Cramer’s V = 0.05), though the difference was small. There were no other significant differences in the actions documented by the GP for the child’s most recent attendance by fee paying status.

**Table 3:** Actions documented by the GP at the most recent consultation in the past year by fee paying status

Action	Fee-paying children (*n* = 1472)	Non fee-paying paying children (*n* = 1631)	*p*-value (Cramer’s V)
Prescribe	918 (62%)	940 (58%)	0.007 (0.05)
Reassure	775 (53%)	889 (55%)	0.30 (0.02)
Refer	205 (14%)	207 (13%)	0.31 (0.02)
Further investigation	167 (11%)	151 (9%)	0.06 (0.03)
Prevention and health promotion advice	129 (9%)	171 (11%)	0.11 (0.003)

## Discussion

### Summary of key findings

This study has important findings relating to attendance rates and outcomes of consultations. Children who are eligible for fee exemption consultations are more likely to have attended their GP in the past year compared to fee-paying children and generate the majority of consultations. Of the children who attended in the past year, those eligible for fee exemption consultations attended more often (median of three consultations compared to two for fee-paying). There is also a subgroup of children eligible for fee exemption consultations who attend very frequently with one quarter attending at least five times per year. In the under sixes, one quarter of those eligible for fee exemption consultations attend at least six times per year. Prescribing and reassurance were the most common actions documented by the GP at the most recent consultation with prevention documented for only one in ten consultations. A higher prescribing rate was seen for fee-paying children compared to those eligible for fee exemption consultations but the difference in rates was small (4%). There were no other significant differences in the actions documented by the GP at the most recent consultation. It is important to note that the methodology did not record socio-economic characteristics. There is a socio-economic bias imbalance in the study, where the group qualifying for free medical care are generally from lower socio-economic backgrounds. Previous research has shown higher attendance rates to general practice among children in this group [19, 20].

### Comparison with the literature

Previously, a study of clinical records from a small number of general practices in one location suggested a much higher attendance rate of children eligible for fee exemption compared to those that are fee paying [21] with attendance rates higher than that previously suggested by self-report, population-based studies [22]. There has been no large scale study of clinical records of children by fee paying status published to date in Ireland. Our finding that those eligible for fee exemption attend more than fee-paying children may be partially explained by the evidence internationally that children of lower income parents are more likely to be brought to the GP with everyday symptoms [19, 23]. Parents who do not have to pay for their children’s consultations may also have a lower threshold for attending. Furthermore, studies using population surveys that facilitated controlling for health status and educational and social levels, have similarly found increased GP visit rates among those eligible for fee exemption compared to fee-paying [24, 25]. A report published in 2017, in which both types of methodologies (patient-reports and clinical records) were assessed, concluded that the real impact of free GP care cannot be estimated by any one of these methodologies alone [26].

The RAND study, a large study of co-payments for health services in the USA from the 1970s, showed that as co-payments for health services increased, the utilisation of the services decreased [27]. Following the introduction of co-payments in Germany in 2004 for outpatient physician visits, a study based on population surveys found that consultation rates reduced, especially in younger and healthier adults. The authors raised concerns for those in lower socio-economic groups [28]. Similarly, an older population-based survey in Ireland showed that co-payments can deter people from consulting their GP, and in particular those from lower socio-economic groups [29]. However, a recent Australian survey showed that for the majority, a small co-payment would not impact on their decision on whether to consult with their GP [30]. This significant finding should stimulate discussion on where the limits of such a co-payment lie.

In Ireland, an increasing demand for GP services coupled with a difficulty in recruiting GPs has created an overstretched work environment [31]. Similarly, in the UK, consultation rates and duration as well as clinical workload has increased indicating that the primary care system is heading for saturation [32]. In this context, General Practice service planning requires urgent attention to ensure that the patients most in need of appointments have access to them. It does not seem unreasonable to suggest that co-payment may have a role in reducing consultation rates with GPs. In fact, a small study conducted in eight general practice clinics found a rise of 9.4% in daytime attendance and 28.7% in out of hours attendances in the year after introduction of free care for children under six years [33]. A systematic review of 47 studies reported that when co-payment reduces consultation rates with GPs and reported no corresponding increase in hospitalisations as a result of co-payment [34]. The same study found that the categories of patients that reduced their consultations the most were lower income and those in need of care so these patients should be exempt from co-payment. If co-payments are to continue, a fine balance must be struck to ensure that patients who need care are not deterred but at the same time are not denied access because of unnecessary appointments. A solution may be to provide a free annual health check appointment for prevention with further appointments involving a small co-payment.

### Outcomes of consultations

In terms of consultation outcomes, reassurance was documented in over half of the consultations and prescribing was the most common action. GPs are responsible for most antibiotic prescribing to children [35]. The slightly higher prescribing rate observed in the fee-paying group may be explained by more pressure to prescribe perceived by GPs from parents who are paying for the consultation or it may be that possibly those parents tend to present their children when the symptoms are more advanced. In Scotland, when incentives for doing health checks on children were removed the rate of uptake decreased significantly [36]. It will be interesting to see if incentivising in Ireland will strengthen the GP role in preventive health care.

### Strengths and limitations of the study

This is the first, large multi-practice study of clinical records comparing consultations of children by fee paying status in Ireland. Participation among the GP network was high (89%), yielding consultation data on over six thousand children. It should be acknowledged, however, that practice management systems are designed and used for administrating the practice rather than research and there may be differences in how information was stored and accessed across practices. Practices also vary in how up to date their patient lists are e.g. removing patients known to have moved away or accurately classifying temporary visitors in the practice management system. The frequency and type of disease coding varies within general practice in Ireland so reliable information on the prevalence of chronic diseases in children is difficult to obtain from practice management systems, as well as employment status of parents and socio-economic group. A significant proportion of paediatric consultations in general practice occur out of hours and these were not included in our study. The socioeconomic bias in the methodology described above is an important limitation.

### Implications for future research and practice

Practice-based networks have been termed “indispensable for the maintenance and development of general practice as an academic discipline” [37]. The GP network that provided the setting for this study has yielded data on consultations involving children in general practice at a very important juncture for child health in Ireland. The years after health care restructuring will present a research opportunity to measure the effect of on help-seeking behaviour and cost to the state free care on attendances, outcomes and preventive health for children in general practice. Although a before and after comparison was beyond the scope of this study, the findings will add to the growing body of knowledge on the potential impact of fees on healthcare utilisation for countries considering co-payment options. A largescale study of attendances to general practice comparing rates before and after the introduction of free GP care for children under six years would be opportune at this point. Considering the UK and Netherlands as a paradigm, where large health care payment reforms led to unexpected health spending costs, any shift towards universal health care be preceded by careful planning with involvement of representatives from General Practice [6].

## Conclusions

Our findings show that, based on general practice records in Ireland, childhood consultation rates are higher in the non-fee-paying category of patients. However, the consultation outcomes for fee-paying and non-fee-paying groups are similar.

## Abbreviations

GMS: General Medical Services
GP: General Practitioner

## References

[1] Saxena S (2010). Primary care management of acute illness in children. London J Prim Care.

[2] Hippisley-Cox J, Vinogradova Y. Final report to the NHS information Centre and Department of Health. Trends in consultation rates in general practice 1995/1996 to 2008/2009: analysis of the QResearch® database. London: NHS Information Centre for Health and Social Care 2009. http://content.digital.nhs.uk/catalogue/PUB01077/tren-cons-rate-gene-prac-95-09-95-09-rep.pdf. Accessed 20 Sept 2017.

[3] Hollinghurst S, Gorst C, Fahey T, Hay AD (2008). Measuring the financial burden of acute cough in pre-school children: a cost of illness study. BMC Fam Pract.

[4] Irish Medical Council. Back to Publications Medical Workforce Intelligence Report 2016 [Internet]. Ireland: Irish Medical Council; 2016. Available from: https://www.medicalcouncil.ie/News-and-Publications/Reports/Medical-Workforce-Intelligence-Report-2016-.html.

[5] The Nuttfield Trust. Number of general practitioners per 1,000 population [Internet]. United Kingdom: The Nuttfield Trust; 2014. Available from: https://www.nuffieldtrust.org.uk/chart/number-of-general-practitioners-per-1-000-population. Kroneman M. Paying General Practitioners in Europe [Internet]. The Netherlands: NIVEL, Netherlands Institute for Health Services Research; 2011. Available from: https://www.nivel.nl/sites/default/files/bestanden/Rapport-paying-gp-in%20europe.pdf.

[6] Toop L, Jackson C (2015). Patient co-payment for general practice services: slippery slope or a survival imperative for the NHS?. Br J Gen Pract.

[7] Brill D (2014). Charging Australians to see a family doctor would make them “think twice,” says think tank. BMJ.

[8] Detollenaere J, Hanssens L, Vyncke V, De Maeseneer J, Willems S (2017). Do we reap what we sow? Exploring the association between the strength of European primary healthcare systems and inequity in unmet need. PLoS One.

[9] British Medical Association. International models of general practice [Internet]. United Kingdom: British Medical Association; 2018. Available from: https://www.bma.org.uk/collective-voice/policy-and-research/nhs-structure-and-delivery/primary-and-community-care/international-models-of-general-practice.

[10] Azzopardi-Muscat N, Buttigieg S, Calleja N, Malta MS (2017). Health system review. Health Syst Transit.

[11] Pullicino G, Sciortino P, Calleja N, Schäfer W, Boerma W, Groenewegen P (2015). Comparison of patients’ experiences in public and private primary care clinics in Malta. Eur J Pub Health.

[12] Department of Health (2016). Health in Ireland key trends 2016 [internet].

[13] Health (General Practitioners Service) Act In. Ireland; 2014. http://www.irishstatutebook.ie/eli/2014/act/28/enacted/en/html. Accessed 20 Sept 2017.

[14] Department of Health. Healthy Ireland – A Framework for Improved Health and Wellbeing 2013–2025: Department of Health. 2013. http://health.gov.ie/wp-content/uploads/2014/03/HealthyIrelandBrochureWA2.pdf. Accessed 20 Sept 2017.

[15] National Association of General Practitioners. 2015. http://nagp.ie/why-gps-oppose-free-care-for-children-under-6/. Accessed 20 Sept 2017.

[16] Jones D, Loader N (2016). Should patients pay to see the GP?. BMJ.

[17] Irish College of General Practitioners. 2017. http://www.icgp.ie/go/library/catalogue/item/6C93C3BF-D93B-568D-4FD67697E1C4C539. Accessed 20 Sept 2017.

[18] Hofmans-Okkes IM, Lamberts H. The international classification of primary care (ICPC): New applications in research and computerbased patient records in family practice. Fam Pract. 1996;13:294–302. 10.1093/fampra/13.3.294 8671139

[19] Saxena S, Majeed A, Jones M (1999). Socioeconomic differences in childhood consultation rates in general practice in England and Wales: prospective cohort study. BMJ.

[20] Edwards A, Pill R (1996). Patterns of help-seeking behaviour for toddlers from two contrasting socio-economic groups: new evidence on a neglected topic. Fam Pract.

[21] Behan W, Molony D, Beame C, Cullen W (2014). Does eliminating fees at point of access affect Irish general practice attendance rates in the under 6 years old population? A cross sectional study at six general practices. Ir Med J.

[22] Behan B, Molony D, Beame C, Cullen W. Are Irish adult general practice consultation rates as low as official records suggest? A cross sectional study at six general practices. Ir Med J 2013;106(10):297–299. 24579407

[23] Beale N, Peart C, Kay H, Taylor G, Boyd A, Herrick D (2010). ‘ALSPAC’infant morbidity and council tax band: doctor consultations are higher in lower bands. Eur J Pub Health.

[24] Nolan A, Layte R. Child Access to GP Services in Ireland: Do user fees matter? 215. http://www.esri.ie/growing-up-in-ireland/growing-up-in-ireland-official-publications-from-the-child-cohort/. Accessed 20 Sept 2017.

[25] Nolan A, Nolan B (2008). Eligibility for free GP care,“need” and GP visiting in Ireland. Eur J Health Econ.

[26] Gorecki P. The Impact of Free GP Care on the Utilisation of GP Services in Ireland: An Evaluation of Different Approaches. https://www.esri.ie/publications/the-impact-of-free-gp-care-on-the-utilisation-of-gp-services-in-ireland-an-evaluation-of-different-approaches/. Accessed 20 Sept 2017.

[27] Gruber J. The role of consumer copayments for health care: lessons from the RAND health insurance experiment and beyond: Citeseer. 2006. http://citeseerx.ist.psu.edu/viewdoc/download?doi=10.1.1.466.5781&rep=rep1&type=pdf. Accessed 20 Sept 2017.

[28] Rückert IM, Böcken J, Mielck A (2008). Are German patients burdened by the practice charge for physician visits ('Praxisgebuehr')? A cross sectional analysis of socio-economic and health related factors. BMC Health Serv Res.

[29] O'Reilly D, O'Dowd T, Galway KJ, Murphy AW, O'Neill C, Shryane E (2007). Consultation charges in Ireland deter a large proportion of patients from seeing the GP: results of a cross-sectional survey. Eur J Gen Pract.

[30] Bingham AL, Allen AR, Turbitt E, Nicolas C, Freed GL (2015). Co-payments and parental decision making: a cross-sectional survey of the impact on general practice and emergency department presentations. Aust Fam Physician.

[31] Teljeur C, Thomas S, O'Kelly F, O'Dowd T (2010). General practitioner workforce planning: assessment of four policy directions. BMC Health Serv Res.

[32] Hobbs FR, Bankhead C, Mukhtar T, Stevens S, Perera-Salazar R, Holt T, Salisbury C, Salisbury C (2016). National Institute for Health Research School for Primary Care Research. Clinical workload in UK primary care: a retrospective analysis of 100 million consultations in England, 2007–14. Lancet.

[33] O’Callaghan ME, Zgaga L, O’Ciardha D, O’Dowd T (2018). Free children’s visits and general practice attendance. Ann Fam Med.

[34] Kiil A, Houlberg K (2014). How does copayment for health care services affect demand, health and redistribution? A systematic review of the empirical evidence from 1990 to 2011. Eur J Health Econ.

[35] Standing Medical Advisory Committee. The path of least resistance [internet]. Department of Health; 1998. Available from: http://antibiotic-action.com/wp-content/uploads/2011/07/Standing-Medical-Advisory-Committee-The-path-of-least-resistance-1998.pdf.

[36] Wood R, Wilson P (2012). General practitioner provision of preventive child health care: analysis of routine consultation data. BMC Fam Pract.

[37] Schers H, Bor H, van den Hoogen H, van Weel C (2008). What went and what came? Morbidity trends in general practice from the Netherlands. Eur J Gen Pract.

